# Heterogeneity and tumoral origin of medulloblastoma in the single-cell era

**DOI:** 10.1038/s41388-024-02967-9

**Published:** 2024-02-14

**Authors:** Hui Sheng, Haotai Li, Han Zeng, Bin Zhang, Yu Lu, Xixi Liu, Zhongwen Xu, Jing Zhang, Liguo Zhang

**Affiliations:** 1 Department of Biotherapy, Cancer Center and State Key Laboratory of Biotherapy, West China Hospital, Sichuan University, Chengdu, 610041 China; 2 Department of Neurosurgery, West China Hospital, Sichuan University, Chengdu, 610041 China

**Keywords:** Tumour heterogeneity, Sequencing

## Abstract

Medulloblastoma is one of the most common malignant pediatric brain tumors derived from posterior fossa. The current treatment includes maximal safe surgical resection, radiotherapy, whole cranio-spinal radiation and adjuvant with chemotherapy. However, it can only limitedly prolong the survival time with severe side effects and relapse. Defining the intratumoral heterogeneity, cellular origin and identifying the interaction network within tumor microenvironment are helpful for understanding the mechanisms of medulloblastoma tumorigenesis and relapse. Due to technological limitations, the mechanisms of cellular heterogeneity and tumor origin have not been fully understood. Recently, the emergence of single-cell technology has provided a powerful tool for achieving the goal of understanding the mechanisms of tumorigenesis. Several studies have demonstrated the intratumoral heterogeneity and tumor origin for each subtype of medulloblastoma utilizing the single-cell RNA-seq, which has not been uncovered before using conventional technologies. In this review, we present an overview of the current progress in understanding of cellular heterogeneity and tumor origin of medulloblastoma and discuss novel findings in the age of single-cell technologies.

## Background

Medulloblastoma (MB), a malignant embryonic tumor of the developing cerebellum, is one of the most common malignant pediatric brain tumors in the posterior cranial fossa. MB accounts for approximately 25% of pediatric brain tumors, which is an important cause of children mortality [1–3]. Based on transcriptomic, genomic, epigenomic and proteomic profiles, human MBs are classified into four principal subgroups: Wingless (WNT), Sonic Hedgehog (SHH), group 3 and group 4 [1, 4–6]. Each subtype has distinct molecular and clinical signatures [7, 8]. WNT MBs account for around 10% of all MB cases with excellent outcome. The WNT signaling aberrant activation caused by *CTNNB1* mutation is the most prominent feature of WNT MBs [9–12]. SHH MBs occur in about 25% of patients and are characterized by activation of SHH signaling, generally arise from the cerebellum hemispheres and vermis [13–16]. Group 3 and group 4 MBs account for about 60% of MB diagnoses and remain the least understood. The classification of group 3 and group 4 MBs has been confound as a subset of these tumors exhibit overlapping molecular signatures [1, 17, 18].

MBs have great intra- and intertumoral heterogeneity among subtypes and patients. Despite aggressive treatment, the prognosis for MB patients is grim. Those who survive the primary tumor suffer severe side effects and often have tumor relapse [19–22]. It has been proposed that tumor heterogeneity, including malignant cell hierarchy and tumor microenvironment diversity, responsible for the failure of therapy [23–25]. MB tumorigenesis and recurrence are thought to be driven by tumor-initiating cells diversity and their interaction with tumor microenvironment [26–30]. Due to the technical limitations, the heterogeneity of tumor cells and its microenvironment have not been fully understood previously. In the last two decades, the application of single-cell technology has greatly promoted understanding the mechanisms of development and diseases. Concurrently, single-cell RNA-seq (scRNA-seq) has been widely used to discover the intra- and intertumoral heterogeneity of central nerve system, including pediatric brain tumors [31–37]. Utilizing of the scRNA-seq technology has revealed novel insights for the tumorigenesis and heterogeneity of tumor microenvironment, which has not been uncovered before using conventional technologies, such as bulk profiling. Here, we review recent studies utilizing single-cell based technologies to explore the malignant cell hierarchy, tumor origin diversity and tumor microenvironment heterogeneity of MBs.

### Molecular classification Of MB

Histological features and multi-omics data have precisely classified MB into four molecular subgroups, including WNT, SHH, group 3 and group 4 [38–40]. Each subgroup has distinct clinical characteristics, genetic aberrations and prognoses (Table 1). WNT MBs account for about 10% of cases with a favorable prognosis and are characterized by activation of the WNT signaling pathway. The most frequently mutated gene in WNT MBs patients is *CTNNB1*, which promotes stabilization and nuclear localization of β-catenin and activation of WNT signaling pathway [10, 41–44]. Other frequently mutated genes are *DDX3X*, *SMARCA4*, *CREBBP* and *KMT2D*, which encode proteins that can interact with β-catenin [9, 45]. By integrating the DNA methylation pattern and gene expression profiling across an expanded primary samples cohort, Cavalli et al. have classified WNT MBs into two subtypes, WNTα and WNTβ [1]. Although both subtypes have similar survival, WNTα is comprised mainly of children and has ubiquitous monosomy 6. WNTβ is enriched for older patients who are frequently diploid for chromosome 6. SHH MBs account for about 25% of MB patients and are defined by activation of SHH signaling pathway [5, 46]. SHH MBs frequently occur in infants and adult patients and exhibit an average risk. Whereas SHH MBs were considered as high risk with metastasis or *MYCN* amplification and very high risk if harboring *TP53* mutation [47–49]. SHH MBs often contain mutations in genes that activate SHH signaling, such as *PTCH1*, *SMO*, *SUFU* and amplifications of *GLI1* and *GLI2* [50–56]. SHH MBs are more heterogeneous compared to WNT MBs and are divided into four subtypes, SHHα, SHHβ, SHHγ and SHHδ [1]. Among these subtypes, SHHα MBs have the worst prognosis and are enriched for *TP53* mutations and amplifications of *MYCN* and *GLI2* [49, 57, 58]. Infant SHH MBs are mainly distributed across SHHβ and SHHγ with disparate outcomes. SHHδ MBs are primarily occur in adults with favorable prognosis and are enriched for *TERT* promoter mutations [59]. Compared to WNT MBs, which contain an aberrant fenestrated vasculature that permits the accumulation of high levels of intratumoral chemotherapy, SHH MBs have an intact blood-tumor barrier, rendering this tumor impermeable and resistant to chemotherapy [60]. Whereas tumor-specific *Piezo2* knockout in SHH MBs disrupts the blood-tumor barrier, decreases the quiescence of Sox2^+^ MB cells, and enhances MB chemosensitivity [61].

**Table 1:** Summary of MB subgroups.

Subgroup	WNT	SHH	Group 3	Group 4
Prevalence [1, 4, 5, 12, 13]	～10%	～25%	～25%	～40%
Prognosis [1, 4, 5, 12, 13]	Good	Intermediate	Poor	Intermediate
Driver alternation [38–40, 55, 56]	*CTNNB1; DDX3X; SMARCA4; TP53*	*PTCH1; TERT; TP53; SUFU; ELP1; U1 snRNA*	*MYC; GFI1; GFI1B; SMARCA4; OTX2; KBTBD4*	*KDM6A; MYCN; CDK6; PRDM6; CBFA2T2*
Cellular origin [15, 16, 41, 72, 74, 81–83, 90, 95, 96, 102, 103]	MFNs	GNPs NEPs Sox2^+^ cells	NSCs; nascent GlutaCN/UBCs; TCPs	Nascent GlutaCN/UBCs
Anatomical origin [15, 16, 41, 98, 101–103]	LRL of dorsal brainstem	EGL	RL^VZ^ for G3γ RL^SVZ^ for G3 TZ for G3	RL^SVZ^
Malignant cell heterogeneity [71–74]	1. Cell cycle 2. Protein biosynthesis 3. Neuronal differentiation 4. WNT signaling	1. Cell cycle 2. Translation and SHH signaling 3. Neuronal differentiation	1. Cell cycle 2. High level of progenitor state 3. Low level of neuronal-like state	1. Cell cycle 2. Low level of progenitor state 3. High level of neuronal-like state
Mouse model [15, 16, 41, 67, 77–83, 128]	*Blbp-Cre; Ctnnb1*^*+/lox(Ex3)*^*;Tp53*^*+/+*^	1. *Ptch1*^*+/–*^*;Tp53*^*–/–*^ 2. *Atoh1- or hGFAP-Cre;SmoM2* 3. *Atoh1- or hGFAP-Cre;Ptch1*^*lox/lox*^ 4. *NeuroD2: SmoA1* 5. *Lig4*^*–/–*^*;Tp53*^*–/–*^ 6. *Parp1*^*–/–*^*;Tp53*^*–/–*^	1. Co-expression of Myc and DNp53 in stem cells or GNPs 2. Co-expression of Myc and DNp53 in embryonic neural progenitors, GABAergic neuronal progenitors or GNPs 3. Co-expression of Myc and Gfi1 (or Gfi1b) in neural stem cell 4. Co-expression of MycN and mutant p53 in cerebellum neural stem cell (*GTML/Trp53*^*KI/KI*^)	Not available

*MFNs* mossy fiber neuron, *LRL* low rhombic lip, *GNPs* granule neuron progenitors, *GlutaCN* glutamatergic cerebellum nuclei, *UBCs* unipolar brush cells, *NSC* neural stem cells, *NEPs* nestin-expressing progenitors, *TCPs* transitional cerebellum progenitors, *EGL* external granular layer, *RL*^*VZ*^ rhombic lip ventricular zone, *RL*^*SVZ*^ rhombic lip subventricular zone, *TZ* transitional zone, *GTML* glutamate transporter 1-tetracycline transactivator and Tetracycline response element-MycN/Luciferase, *KI* knockin.

Group 3/4 MBs are the most common subgroups and account for about 60% of all MB patients with more complicated pathological and molecular features [9]. Group 3 MBs are considered as the most aggressive subgroup because of the high metastatic potential and the poor survival [5, 12, 17, 62]. Group 3 tumors contain recurrent *MYC* amplifications, *GABRA5* overexpression and *SMARCA4* mutations [7, 63–65]. Due to a lack of unified mutation or activated pathway, group 3 MBs are often clustered based on their transcriptional profile and genomic methylation pattern [66]. Based on the integrated analysis of gene expression and DNA methylation, three group 3 MB subtypes have been identified (G3α, G3β, G3γ) [1]. Most of G3α cases are involved in infants under 3 years and have a frequent Chromosome 8q loss. G3β MBs tumors have a higher frequency of *GFI1* and *GFI1B* activation and *OTX2* amplifications [67, 68]. G3γ MBs have the worst prognosis with harboring *MYC* amplification. Group 4 MBs are the most common form of MB and account for about 40% of all MBs [5]. Similar to group 3, group 4 MBs have no unified molecular signature. The highly prevalent putative driver events in group 4 MBs involve overexpression of *PRDM6*, *GFI1* and *GFI1B*, somatic mutations of *KDM6A*, *ZMYM3*, *KMT2C* and *KBTBD4*, and amplifications of *MYCN*, *OTX2* and *CDK6* [40, 69]. Three subtypes were identified within group 4 MBs [1]. G4α MBs are enriched for *MYCN* amplifications. G4β MBs are enriched for *SNCAIP* duplications and *GFI* activation. Whereas the G4γ MBs are enriched for *CDK6* amplifications, chromosome 8p Loss and 7q gain.

Global proteomic and post-translational modification analysis performed by Archer et al. identified very stable subsets of SHH and group 3 MBs [70]. SHHa MBs contain a higher level of proteins associated with mRNA processing, splicing and transcription, MYC pathway, chromatin remodeling and DNA repair. Whereas proteins with higher levels in SHHb MBs were linked to neuronal and neurotransmitter-like activity. The proteomic features associated with group 3a MBs likely represent the MYC-activated form of MBs, and the proteomic data for group 3b samples represent the known group 3/4 continuum. Furthermore, they revealed that MYC activation by phosphorylation defined a higher risk subset of group 3 patients and inhibiting PRKDC sensitized MYC-activated MBs tumors cells to radiation. MYC-activated group 3 MBs represent one of the most aggressive and poorly understood MBs. Targeting MYC-associated pathways may provide a foundation for future therapeutic strategies. Gwynne et al. utilized the CRISPR-Cas9 loss-of-function screen for a patient-derived MYC-activated group 3 MB cell line and uncovered that DHODH sustained the transcriptional activity of c-Myc and drove cell-cycle progression in *MYC*-amplified group 3 MBs [71]. DHODH inhibitors exerted on-target therapeutic effects by altering the metabolome and lipidome of MYC-amplified group 3 MBs in a uridine-dependent manner.

### Heterogeneity of malignant cells in human MB

**Fig. 1:**
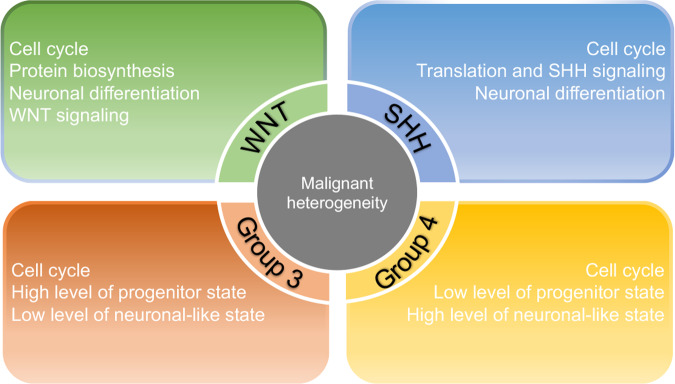
Heterogeneity of malignant cells for each MB subgroup. NMF analysis identified the intratumoral heterogeneity with distinct programs for each MB subgroup.

MBs exhibit well-characterized intertumoral heterogeneity among subtypes and patients, which has been wildly characterized by multi-omics profiling studies. However, the intratumoral heterogeneity of MB was rarely studied until the appearance of single-cell technologies. Recent scRNA-seq studies have uncovered the intratumoral cellular heterogeneity and putative hierarchies at single-cell resolution for all four subgroups (Fig. 1). Each subgroup malignant tumor cells were committed to distinct neuronal lineages of the developing cerebellum along varying degrees of differentiation. Hovestadt et al. performed full-length scRNA-seq to profile MB malignant cells including all four subgroups and used non-negtive matix factorization (NMF) method to uncover the malignant cell heterogeneity [72]. Four metaprograms were identified for WNT MBs, including cell-cycle activity, protein biosynthesis and metabolism, neuronal-like differentiation and WNT pathway. Scoring each cell with four metaprograms revealed that cell-cycle activity was restricted to cells high for genes associated with protein biosynthesis and metabolism, but lower for neuronal differentiation. Jessa and colleagues identified three major cell populations for WNT MBs, including two nonproliferating subpopulations with different WNT activity and an early neuronal-committed subpopulation [73]. Both studies identified malignant cell populations with WNT signal activation, consistent with the driver mutation of *CTNNB1* in this MB subgroup. For SHH MBs, two paralleled studies demonstrated that malignant cells are most similar to granule neuron progenitors (GNPs) lineage and with various differentiation stages [72, 74]. Hovestadt et al. divided malignant cells into three transcriptional programs, which contain markers of cell-cycle activity, translational associated genes and neuronal differentiation [72]. Comparation of the metaprograms to mouse cerebellum cell populations revealed that malignant cells correlated with different developmental stages of GNPs lineage. Furthermore, SHH MBs malignant cells can be separated into two age-associated categories. The infant tumors correlated with intermediate and mature granule neurons, whereas adult tumors correlated with undifferentiated progenitors. Vladoiu et al. demonstrated that the SHH MBs scRNA-seq clusters are mostly resembled to GNPs lineage [74]. SHH MBs contain a variety of tumor cell types that represent different stages of GNPs differentiation and that might exhibit distinct clinical behaviors and therapeutic responses [75].

Group 3 and group 4 MBs account for around 60% of MB diagnoses with more complicated pathological and molecular features. The bulk RNA-seq studies have indicated a subset of tumors exhibiting overlapping molecular signatures that confused the classification between these two subtypes. They have least understood of tumorigenesis and cellular heterogeneity than WNT and SHH subtypes. Hovestadt et al. identified three distinct transcriptional programs contained markers of cell-cycle program, undifferentiated progenitor-like program as well as differentiated neuronal-like program for both group 3 and group 4 MBs [72]. Scoring each group 3/4 MBs cell for these programs revealed prototypic group 3 tumors were dominated by the undifferentiated progenitor-like program, whereas the differentiated neuronal-like program was observed in almost all cells from prototypic group 4 tumors, consistent with the neuronal differentiation phenotype for group 4 tumors. Of note, lacked neuronal differentiation cells were observed in group 3 MBs with *MYC* amplifications, indicating that oncogenic *MYC* expression may potentiate an undifferentiated progenitor-like state. Whereas the group 3/4 intermediate tumors comprised a mixture of undifferentiated and differentiated programs. By deconvoluting of human group 3/4 MBs against mouse cerebellum cell populations, Vladoiu et al. demonstrated the group 3/4 MBs resemblant to neuronal lineages of the developing cerebellum along varying degrees of differentiation [74]. Group 3 MBs cell clusters exhibit highly divergent lines of normal cerebellum in the GNPs, unipolar brush cells (UBCs), Purkinje cells and GABAergic interneuron lineages, which reflects an origin from uncommitted cerebellum stem cells, followed by partial differentiation of transformed cells along diverse developmental lineages. group 4 MBs cell populations transcriptionally mirror the differentiated UBCs, UBC progenitors and GNPs lineage, consistent with a model in which group 4 MBs arises from a bipotential progenitor cell population, which can rise to both the GNP and the UBC lineages.

The study performed by Riemondy et al. obtained similar conclusion of the malignant cellular heterogeneity for the human MB subgroups [76]. Six subpopulations of neoplastic cells were identified in SHH tumors including two cell cycle, two progenitor, and two neuronally differentiated subpopulations. The differentiated cell subpopulations are significantly corelated with favorable outcome for survival. Consistent with Hovestadt et al.’s conclusion, the SHH progenitor populations were highest enriched in non-infant SHH MB subtypes. For group 3 MBs, five cell subclusters were identified that included two differentiated populations, two progenitor populations and one mitotic population. Notably, the two progenitor populations were enriched of *MYC* activity and indicated poor outcomes. Six major neoplastic subpopulations were identified in group 4 samples, including three cell cycle, two progenitor and one differentiated subpopulation, which were with some degree of similarity with subpopulations identified in group 3 MBs.

### Heterogeneity of malignant cells in animal MB

The animal models have been widely used to study the mechanism for MB tumorigenesis in last several decades [15, 16, 41, 67, 77–83]. Recently, several studies have uncovered the malignant cell heterogeneity in MB animal models. Zhang et al. have performed scRNA-seq to profile cellular heterogeneity of SHH MB mouse model [84]. Consistent with previous studies, GNPs populations accounted for most of the analyzed transcriptomes. The GNPs populations were subdivided into mitotic proliferating cells, which could be further differentiated by phases of the cell cycle and mature postmitotic populations. The malignant cell populations in SHH MBs that mirror GNP lineage development in the cerebellum. Ocasio et al. used scRNA-seq and lineage tracing to analyze cellular diversity in SHH MB mouse model [85]. The tumor cells and stromal cells showed either a spectrum of neural progenitor-differentiation states or glial and stem cell markers. After treatment with vismodegib, a SHH signaling inhibitor, the *Hes1*-expressing tumor cells were changed from proliferative state to differentiated state. However, *Myod1*-expressing tumor cells were vismodegib-resistant and remained proliferative. The tumor cell heterogeneity, identified by scRNA-seq for SHH inhibitor response, can explain the clinical drug resistance and relapse after targeted inhibitor therapy. Two scRNA-seq studies profiled tumor cells heterogeneity using *Ptch1*+*/−* mice model. Cheng et al. identified three tumor cell subpopulations, including dividing tumor cells, quiescent tumor cells and more differentiated tumor cells [86]. Tumor cells resemble the cerebellum neuronal progenitors and the differentiated tumor cells permanently lose their tumorigenic capacity. Further studies demonstrated that enhanced expression of *NeuroD1* by treatment with EZH2 Inhibitors can induce MB cells differentiation and prevent tumor progression. Luo et al. demonstrated the transformed granule cells (GCs) in MB closely resemble developing granule neurons of varying differentiation states [87]. However, transformed granule neuron progenitors in MB exhibited less tendency to differentiation compared with cells in normal development.

To uncover the differences between SHH MBs derived from progenitors (*Math1-Cre/SmoM2*) or stem cells (*hGFAP-Cre/SmoM2*) at single-cell resolution [88–90], Malawsky et al. profiled these two animal models and identified tumor cell subpopulations in a range of states that paralleled GNPs development, from proliferative cells to non-proliferative cells at different stages of neural differentiation [91]. Although tumor cell subpopulations were similar between these two models, stem cell-derived MBs progressed faster, contained more Olig2-expressing stem-like cells and showed radiation-resistance. Riemondy et al. assessed the cellular heterogeneity using two group 3 allograft mice models (MP, overexpression of *Myc* and dominant-negative *Trp53*; MG, co-expression of *Myc* and *Gfi1*) and one SHH transgenic mouse model (mutant Smo activated in the *Atoh1* lineage) [67, 76, 82, 92, 93]. They identified subpopulations in mouse MB models corresponding to human subgroup-specific subpopulations.

### Heterogeneity of cellular origins for MB

MBs have distinct cellular origins for each subgroup. Previous bulk-profiling studies have explored the cellular origins of each MB subgroup used animal models. The cellular origins of WNT and SHH MBs have been more interpreted than that of group 3/4 MBs due to the more complex of the latter two subtypes and unavailable of animal model. Gibson et al. have revealed that WNT MBs were derived from outside the cerebellum and cells of the dorsal brainstem [41]. Genes marking human WNT MBs are more frequently expressed in the embryonic dorsal brainstem and lower rhombic lip (LRL) than that in the upper rhombic lip (URL) of developing cerebellum [94]. Two studies parallelly performed by Schuller et al. and Yang et al. indicated that SHH MBs originated from GNPs after aberrant activation of the SHH pathway [15, 16]. Li et al. demonstrated that nestin-expressing progenitors (NEPs) resided in the deep part of the external granular layer (EGL) are also as a cellular origin for SHH MBs [95]. Aberrant activation of SHH signaling in NEPs exhibited more severe genomic instability and gave rise to SHH MBs more efficiently than GNPs. Vanner et al. revealed that quiescent Sox2^+^ cells derived tumor growth and responded for relapse in SHH MBs [96]. One study performed by Selvadurai et al. indicated that aberrant activation of SHH signaling in the transient stem-like Sox2^+^ cells within EGL layer caused persistent hierarchical growth and led to SHH MBs [90]. Three studies performed by Pei et al., Kawauchi et al. and Swartling et al. have demonstrated that group 3 MBs may derived from cerebellum neural stem cells by overexpressing *Myc* and mutant *Trp53* in stem cells and orthotopic transplantation [81, 82, 97]. Kawauchi et al. have used in utero electroporation method to demonstrate that group 3 MBs can be developed in situ from different multipotent embryonic cerebellum progenitor cells via conditional expression of *Myc* and loss of *Trp53* function in several Cre-driving mouse lines [83].

**Fig. 2:**
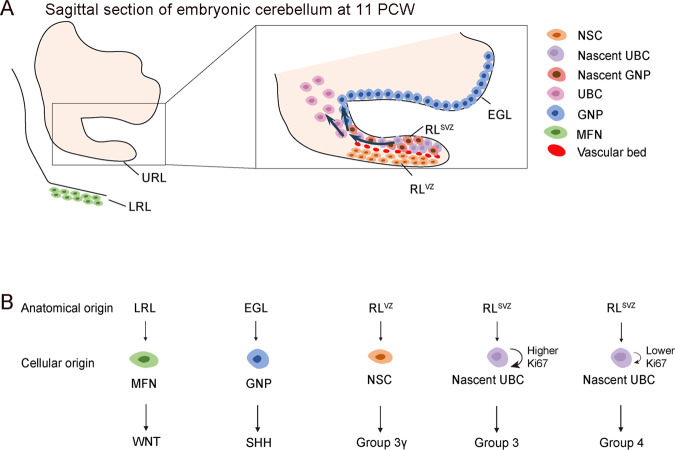
Cellular and anatomical origins of MB subgroups. **A** Schematic representation of sagittal section of the developing embryonic cerebellum at 11 PCW showing the location of the precursors that give rise to the distinct MB subgroups shown in (**B**). **B** WNT MBs derive from MFNs in the dorsal brainstem, SHH MBs derive from GNPs in the EGL, group 3 MBs originate from stem cells and nascent UBCs in RL^VZ^ and RLS^VZ^, respectively, group 4 MBs originate from nascent UBCs in RL^SVZ^. MFNs mossy fiber neuron, GNPs granule neuron progenitors, UBCs unipolar brush cells, EGL external granular layer, LRL lower rhombic lip, URL upper rhombic lip, RL^VZ^ rhombic lip ventricular zone, RL^SVZ^ rhombic lip subventricular zone.

Although the cellular origins of MBs have been revealed to some extent using conventional technologies, it still needs to be comprehensively studied due to the marked heterogeneity of tumor-initiating cells. Single-cell technologies provide powerful tools for accurately identifying the cellular origins (Fig. 2). For WNT MBs, LRL progenitors in the embryonic dorsal brainstem have been implicated as the potential cellular origin [41]. However, the precise cell lineage has not yet been defined due to shared expression of markers between auditory LRL and pre-cerebellum LRL-derived lineages. By deconvolution of WNT MBs bulk transcriptomic data to mouse developmental pons/hindbrain and the forebrain single-cell data, Jessa et al. found expression of WNT MBs marker genes was restricted to a pontine mossy fiber neuron (MFN) population and the MFN lineage was the best match for WNT MBs [73]. scRNA-seq profiling of three WNT MBs patient samples uncovered that tumor cell clusters best matched to normal developmental MFN cell population at single-cell level, suggesting the MFN lineage as the cellular origins of WNT MBs. However, the study performed by Hovestadt et al. revealed that this cellular origin of WNT MBs was not evident as it was failed to identify significant correlation between WNT MBs single-cell programs and cerebellar cell populations, potentially due to incompleteness of reference atlases or extracerebellar origin for WNT MBs [72]. Thus, the utilization of human embryonic cerebellum and hindbrain single-cell populations as a reference atlas may provide more convincing evidence for the cellular origin of WNT MBs. Okonechnikov et al. generated an extensive single-nucleus RNA-seq (snRNA-seq) dataset of human development cerebellum as reference atlas that covered a wide range of cell states [98]. Using this reference atlas, they confirmed that majority of SHH MBs were corresponded to GNPs and postmitotic GCs, whereas group 3/4 resembled GC/UBC progenitors and early differentiating UBCs. Moreover, *MYC/MYCN* amplifications can derive group 3/4 tumor cells away from the original GC/UBC lineage and exhibited worse outcome.

By deconvolution of bulk RNA-seq and single-cell data of each MB subgroup to mouse developing cerebellum scRNA-seq cell clusters, two parallel studies have deeply explored the cellular and anatomical origin of each MB subgroup. Vladoiu et al. profiled tumors of the SHH, group 3 and group 4 subgroups, demonstrating subgroup-specific resemblance to distinct neuronal lineages of the developing cerebellum along varying degrees of differentiation and were similar to specific time points during fetal life [74]. By deconvoluting bulk RNA-seq transcriptomes of human MBs against defined scRNA-seq cell clusters of mouse cerebellum, they demonstrated that SHH MBs share most similarities to GNPs clusters, as supported by previous experimental studies. Comparison of group 3 MBs to developmental cerebellum cell clusters revealed a resemblance between group 3 MBs to Nestin-expressing cerebellum early stem cells. Group 4 MBs was transcriptionally best matched to cells of the UBC lineage, which are glutamatergic interneurons derived from the URL. Furthermore, deconvolution of scRNA-seq malignant cell clusters of human MBs against the cell clusters of mouse cerebellum revealed that SHH MBs scRNA-seq cell clusters remained most similar to cells in the GNPs lineage, suggesting this subgroup MBs origin from GNPs. Group 3 MBs scRNA-seq cell clusters were similar to multiple developmental normal lineages, suggesting its origin from an early uncommitted cerebellum stem cell, followed by partial differentiation along diverse developmental lineages. Group 4 MBs scRNA-seq cell subpopulations mirrored UBCs at different development time points and were predominantly similar to *Calb2*-expressig UBCs, as well as GNPs lineage [99]. This data is consistent with a model in which group 4 MBs arise from a bipotential progenitor cell population that can give rise to both UBC and GNPs lineages. Hovestadt et al. obtained the similar conclusion of tumor origin for group 4 MBs [72] and revealed both UBCs and glutamatergic cerebellum nuclei (GlutaCN) were highly correlated with group 4 MBs expression pattern. The UBC and GlutaCN markers were specifically expressed in group 4 malignant cells, which implicated UBCs and GlutaCNs of the embryonic cerebellum as candidate cell-of-origin for group 4 MBs.

The cellular origins of MB also have been studied by scRNA-seq in animal model. Zhang et al. demonstrated a developmental hierarchy of progenitor pools in SHH MBs and identified OLIG2-expressing glial progenitors as transit amplifying cells at the tumorigenic onset and during recurrence [84]. Although OLIG2^+^ progenitors become quiescent stem-like cells in full- blown tumors, they are highly enriched in therapy-resistant and recurrent MBs. Depletion of mitotic Olig2^+^ progenitors or *Olig2* ablation impeded tumor initiation, indicating that glial lineage-associated OLIG2^+^ progenitors are cellular origin of SHH MBs and OLIG2-driven oncogenic networks as potential therapeutic targets.

### Heterogeneity of anatomical origins for group 3/4 MB

The developing human RL displays specific features compared to other mammals, which is splited into the RL ventricular zone (RL^VZ^) and the RL subventricular zone (RL^SVZ^) around 11 post-conception weeks (PCW) [100]. RL^VZ^ is primarily composed of stem cells, whereas the RL^SVZ^ is primarily composed of proliferative progenitor cells, including nascent UBCs and nascent GNPs. However, this subcompartmentalization is short-lived and no longer visible following RL internalization at 14 PCW. RL produces more GNPs at early stage and decreased production of GNPs after 11 PCW. In contrast, production of early UBCs is increased after 14 PCW and throughout human gestation. The spatiotemporally expanded pool of MB-susceptible UBC progenitors provides a statistically larger risk for group 3/4 MBs tumorigenesis (Fig. 2).

Two parallel studies performed by Hendrikse et al. and Smith et al. used human development cerebellum scRNA-seq data as a reference to deconvolute the human MBs cell subpopulations and revealed new insights for cellular and anatomical origins of group 3/4 MBs [101, 102]. Both studies uncovered that RL is the unified anatomical origin of group 3 and group 4 MBs and failure of human RL differentiation underlies these two subgroup MBs formation. Hendrikse et al. demonstrated that group 4 MBs driver mutations are enriched in the core binding factor alpha (CBFA) complex and genes encoding the components of this complex expressed early in the progenitor cells within the RL^SVZ^, indicating the RL^SVZ^ is the anatomical origin of group 4 MBs [102]. In addition, the scRNA-seq transcriptional comparison between the developing human cerebellum and MBs cells revealed that group 3/4 MBs cells were most similar to the RL^SVZ^, whereas the more deadly group 3 gamma subtype (G3γ) displayed enrichment for the earlier RL^VZ^. Group 3/4 MBs displayed a differentiation block and group 3 MBs displayed the lowest similarity to normal cerebellum cells. Group 4 MBs and some group 3 MBs tumors arise in the RL^SVZ^ owing to the specific human RL split. Consistent with Hendrikse’s studies, Smith and colleagues demonstrated that cellular and anatomical origins of group 3/4 MBs tumors are mapped to the RL^SV^ and to GlutaCN or UBC lineages [101]. Gene sets that defined group 3/4 MBs were enriched in GlutaCN/UBCs. The early progenitors of the GlutaCN/UBC lineage were classified as group 3-MB-like, whereas more differentiated cells were classified as group 4-MB-like, suggesting that group 3/4 MBs tumor cells align with GlutaCN/UBC-lineage-committed progenitors of the RL^SVZ^ and are defined by the extent of their differentiation.

Since the diversity of precursor cells in the developing cerebellum primordia, MBs may have multiple cellular and anatomical origins, especially for the most aggressive group 3 MBs, which are lack of unified mutation or activated signaling pathway. Luo et al. identified a unique transitional cerebellum progenitor (TCP) as a putative cellular origin for aggressive MBs, such as group 3 MBs [103]. TCPs are localized in the cerebellum ventricular zone (VZ), RL transitional zone (TZ) and RL^VZ^ region rather than RL^SVZ^ region. TCPs are increased from PCW 9 to PCW 12 but reduced progressively beginning at PCW 14. Trajectory analysis identified TCPs as a precursor to generate GCP, UBC and Purkinje lineage cells. Comparison to fetal cerebellar cell population profiles revealed that group 3 MBs exhibited the strongest similarity to human fetal TCPs, followed by UBC-lineage cells. Whereas the SHH MBs and group 4 MBs showed similarity with GNPs and UBCs respectively. In addition, the TCP-like cells were present in higher abundances in group 3 MB than in group 4 and SHH MBs. The unique tumor-driver networks and enhancer-hijacking events correlated with *MYC* activation were identified in TCP-like cells, pointing to the putative cellular origin of group 3 MBs and the potential therapeutic avenues.

### Heterogeneity of immune microenvironment

The tumor microenvironment is one of the most important factors for tumor progression and treatment response in many cancers [104–108]. Therapeutic targeting of the interaction between tumor and microenvironment is the most promising treatment method. Tumor-infiltrating immune cells, principally lymphocytes and myeloid cells, have been shown to be of prognostic relevance and predictive for response to chemotherapy in various tumors [104, 109–113]. Previous studies have described the characteristics of immune microenvironment of MBs by conventional methods. Margol et al. revealed SHH MBs had significantly higher infiltration of tumor-associated macrophages (TAMs) than that in the group 3/4 subgroups [114]. The interactions of TAMs and SHH MB cells may contribute to tumor growth revealing TAMs as a potential therapeutic target. Pham et al. characterized immune infiltrating cells and observed significantly higher percentages of dendritic cells, infiltrating lymphocytes, myeloid-derived suppressor cells and TAMs in murine SHH MBs compared with group 3 MBs [115]. Whereas group 3 tumors had higher percentages of CD8^+^ T cells. Bockmayr et al. showed that SHH MBs displayed strong signatures of fibroblasts, T cells and macrophages, while markers of cytotoxic lymphocytes were enriched in group 4 MBs [116]. Garancher et al. indicated that the failure of MG MBs (co-expression of *Myc* and *Gfi1*) to grow in immunocompetent mice was due to rejection by T cells, whereas tumor necrosis factor could overcome immune evasion in *p53*-mutant MP MBs (overexpression of *Myc* and dominant-negative *Trp53*) [117]. Maximov et al. revealed that TAMs exhibited anti-tumoral properties in SHH MBs [118]. Conversely, Yao et al. indicated that TAMs can be polarized by tumor-derived astrocytes and secrete IGF1 to promote tumor progression [119]. The incomplete understanding of the roles of immune cells are due to the complex diversity of MB immune microenvironment and lacking powerful tool to investigate the microenvironment heterogeneity.

Recent advancements of single-cell technologies enhanced the understanding of tumor immune heterogeneity (Table 2). Riemondy et al. described the landscape of immune cell heterogeneity at single-cell resolution for childhood MBs [76]. Two main clusters of immune cells, lymphocytes and myeloid cells, were identified with variable proportions between individual tumor samples. The myeloid cell proportions were significantly more abundant in SHH MBs than that in group 3/4 MBs. Re-clustered lymphocytes and myeloid cells revealed four lymphocyte clusters, six myeloid cell clusters and one cell-cycle-related cluster.

**Table 2:** Immune cell types and functions in MB subgroups.

References	Organism	Immune cell types	MB subgroups	Key findings
Margol et al. [114]	Human	TAM	WNT, SHH, G3, G4	SHH MBs had significantly higher infiltration of TAMs than that in the group 3/4 MBs
Pham et al. [115]	Mouse	Myeloid cells, lymphocytes	SHH, G3	Higher percentages of DCs, infiltrating lymphocytes, MDSCs and TAMs in murine SHH MBs. Whereas group 3 MBs had more CD8^+^ PD-1^+^ T cells within the CD3^+^ population
Bockmayr et al. [116]	Human	TAM, T cells	WNT, SHH, G3, G4	SHH MBs displayed strong signatures of T cells and TAM, while markers of cytotoxic lymphocytes were enriched in group 4 MBs
Maximov et al. 2019 [118]	Mouse	TAM	SHH	TAMs exhibited anti-tumoral properties in SHH MBs
Yao et al. 2020 [119]	Mouse	TAM	SHH	TAMs can be polarized by tumor-derived astrocytes and secrete IGF1 to promote tumor progression
Dang et al. [115]	Mouse	TAM, T cells, neutrophils	SHH	TAMs are derived from microglia and circulating monocytes. TAMs are increased after radiation along with decreased T cells and neutrophils infiltration
Garancher et al. [117]	Mouse	T cells	G3	Failure of MG MBs to grow in immunocompetent mice was due to rejection by T cells and TNF could overcome immune evasion in p53-mutant MP MBs
Riemondy et al. [76]	Human	Myeloid cells, lymphocytes	WNT, SHH, G3, G4	Myeloid cells and lymphocytes were two main immune cell types identified in MBs and Myeloid cells were more abundant in SHH MBs than that in group 3/4 MBs

*TAMs* tumor-associated macrophages, *DCs* dendritic cells, *MDSCs* myeloid-derived suppressor cells, *IGF1* insulin-like growth factor 1, *MG* co-expression of *Myc* and *Gfi1, MP* overexpression of *Myc* and dominant-negative *Trp53,* *TNF* tumor necrosis factor.

**Fig. 3:**
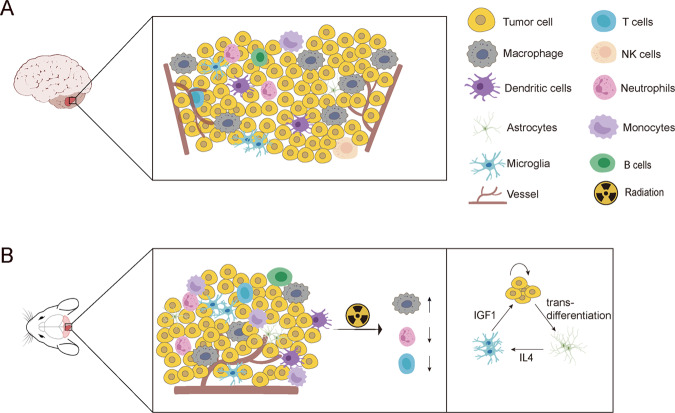
The heterogeneity of immune cell types in MB. **A** Human MBs consist of several distinct immune cell types, including macrophages, microglia, dendritic cells, NK cells, T cells, neutrophils, monocytes and B cells. **B** Mouse MBs consist of several distinct immune cell types, radiation treatment can lead to an increase of tumor-associated macrophages along with decreased of T cells and neutrophils infiltration. Microglia cells within mouse SHH MB can be polarized by tumor-derived astrocytes and secrete IGF1 to promote tumor progression.

The most abundant myeloid subpopulation was named complement myeloid with high expression of complement component 1q subunits. Another myeloid subpopulation was characterized by the expression of markers for anti-inflammatory and M2 myeloid polarization and this population was named M2-activated myeloid. This type of myeloid cells showed the strongest subgroup association, being more abundant in SHH MBs than that in group 3/4 MBs. As exhibiting high expression of microglia markers and low expression of activated myeloid markers, a cell population was named nonactivated microglia with less abundance in SHH MBs compared to group 3/4 subgroups. Two further myeloid subpopulations expressed MHC class II genes were identified with chemokines expression or C-lectins expression. Riemondy et al. revealed that the most MB lymphocytes population was T cells without being able to separate CD4 from CD8 T cells due to the technical resolution of their study. The remaining lymphocyte clusters were natural killer (NK) cells, B cells and regulatory T cells with low abundance. Dang et al. used animal model to reveal the TAMs heterogeneity in SHH MBs [120]. They identified three TAM subpopulations derived from monocytes and two TAM subpopulations were microglia-derived. Further studies revealed an increased number of immunosuppressive monocyte-derived TAM subpopulation after radiation therapy along with decreased T cells and neutrophils infiltration. Thus, compositions of MB microenvironment exhibit dynamic changes with treatment and differ significantly between chemotherapy and radiation therapy (Fig. 3).

### Heterogeneity of spatial transcriptomics of MB

scRNA-seq is a powerful tool that can be used to dissect intra- and intercellular heterogeneity at the single-cell level. It has been widely used to characterize tumor cell subpopulations and their association with tumor microenvironment. However, scRNA-seq alone cannot provide spatial information or the intratumoral spatial heterogeneities. Recently, the spatial transcriptomics sequencing (ST-seq) technology has emerged as a powerful tool to address the limitations of scRNA-seq, providing whole transcriptome analysis across intact tissue sections without dissociation of cells from their in situ localization [121–125]. Vo et al. performed ST-seq to investigate the spatial organization of cells in patient-derived orthotopic xenograft (PDOX) SHH MB sections [126]. ST-seq identified and accurately mapped cell subpopulations across the tumor regions and surrounding cerebellum cortex. Cell subpopulations of oligodendrocytes, purkinje cells, granule neurons and basket/stellate cells were mapped to histologically identifiable regions within surrounding cerebellum cortex. The identified regions within tumor tissue include mouse macrophages, scattered tumor cells and meningeal arteries with corresponding cell subpopulations. Furthermore, the CDK4/6 inhibition treatment with Palbociclib resulted in reduced cellular heterogeneity and led to higher levels of neuronal differentiation within tumors. A transcriptionally distinct interface region was defined where tumors contacted the microenvironment and the tumor cells within this region continued to proliferate despite Palbociclib treatment. The astrocytes and tumor-associated microglia cells were identified as the most abundant cell types within the interface region and the paracrine feedback loop involved in these two cell types may promote the continued proliferation of tumor cells.

## Conclusion

MBs are thought to be resulted from dysregulated reprogramming of normal cerebellum development. Previous bulk RNA-seq and other conventional technologies have discovered the intertumoral heterogeneities among patients and subgroups. However, the intratumoral heterogeneity and cellular origin heterogeneity have not be uncovered until the emergence of single-cell technologies. Recent scRNA-seq studies have steadily increased and changed our perception of this kind pediatric cerebellum tumors (Table 3). Cluster-specific markers and NMF-based classification are two main methods for exploring cellular heterogeneity. The cluster-specific marker-based studies identify subgroup-specific cell subpopulations. Cell cycle, undifferentiated progenitor and neuronal-like differentiation are the three most common programs identified in all four MB subgroups by NMF analysis. WNT MBs are composed of MFN lineage cells, whereas SHH MBs are composed of GNPs lineage with divergent differentiation. Unlike SHH and WNT subgroups, the most common group 3/4 subgroups exhibited more complicated pathological and molecular features. In addition, the bulk RNA-seq studies have indicated a subset of tumors exhibiting overlapping molecular signatures that confused the classification between these two subgroups. scRNA-seq uncovered that group 3/4 tumors were most similar to UBC-like cells with group 3 MBs dominated by the undifferentiated progenitors, whereas group 4 MBs with more differentiated transformed tumor cells. As uncovered by scRNA-seq data, heterogeneity of cellular and anatomical origins has been identified for each MB subgroup. Both GNPs and early undifferentiated progenitors can give rise to SHH MBs. Since there are no well-established mouse models, the tumor origins of group 3/4 subgroups were not fully uncovered. The single-cell technologies provided powerful tools to uncover the tumor origins for group 3/4 subgroups. scRNA-seq reveals that the RL^SVZ^ is the converged region for group 3/4 subgroups anatomical origin and failure of human rhombic lip differentiation underlies group 3/4 MBs formation. The human specific feature of RL^SVZ^ is one of the most critical reasons for delayed discovery of cellular and anatomical origins for group 3/4 MBs.

**Table 3:** Single-cell studies for MB discussed in this review.

References	Organism	Assay	Tissues profiled	Accession	Key findings
Hovestadt et al. [72]	Human Mouse	scRNA-seq	WNT, SHH, G3, G4, Mouse cerebellum	GSE119926; PRJEB23051	Heterogeneity of malignant cells for each subtype and GlutaCN/UBC as candidate cell-of-origin for Group 4 MBs.
Jessa et al. [73]	Human Mouse	scRNA-seq snRNA-seq	WNT, Mouse brain	EGAS00001003368; GSE133531; GSE133531	Heterogeneity of malignant cells for WNT MBs and MFN lineage as the cellular origins of WNT MBs
Vladoiu et al. [74]	Human Mouse	scRNA-seq	SHH, G3, G4, Mouse cerebellum	GSE118068; EGAD00001004435	Subgroup-specific resemblance of tumor cells to neuronal lineages of the developing mouse cerebellum
Riemondy et al. [76]	Human Mouse	scRNA-seq	WNT, SHH, G3, G4, Mouse model of SHH and G3	GSE156053	Heterogeneity of malignant cells and immune cells landscape in each subgroup
Zhang et al. [84]	Mouse	scRNA-seq	Mouse model of SHH	GSE120974	Identification of OLIG2-expressing glial progenitors as transit amplifying cells at the tumorigenic onset and during recurrence
Ocasio et al. [85]	Mouse	scRNA-seq	Mouse model of SHH	GSE129730	SHH MB intratumoral heterogeneity and mechanism of SHH inhibitor resistance
Cheng et al. [86]	Mouse	scRNA-seq	Mouse model of SHH	GSE150752	SHH MB intratumoral heterogeneity and enhanced expression of NeuroD1 by treatment with EZH2 Inhibitors can induce MB cells differentiation
Malawsky et al. [91]	Mouse	scRNA-seq	Mouse model of SHH	GSE150579	Stem cell-derived MBs progress faster, contain more Olig2-expressing stem-like cells and exhibit radiation-resistance
Luo et al. [87]	Mouse	scRNA-seq	Mouse model of SHH	GSE156633	Transformed GNPs in SHH MB resemble developing granule neurons of varying differentiation states.
Hendrikse et al. [102]	Human Mouse	scRNA-seq snRNA-seq	SHH, G3, G4, G3 cell lines, Human cerebellum	EGAS00001005826; GSE189238; GSE200791	Human specific split of RL to RL^VZ^ and RL^SVZ^ and Group 4/3 MBs tumors arise from UBC progenitors within the RL^SVZ^
Smith et al. [101]	Human	scRNA-seq snRNA-seq	SHH, G3, G4, human cerebellum, human cerebellum organoids	GSE207266	Human specific split of RL to RL^VZ^ and RL^SVZ^, Group 3/4 MBs tumor cells arise from GlutaCN/UBC progenitors of the RL^SVZ^ and are defined by the extent of their differentiation.
Okonechnikov et al. [98]	Human	scRNA-seq snRNA-seq	SHH, G3, G4, human cerebellum	www.brain-match.org	Subgroup-specific resemblance of tumor cells to neuronal lineages of the developing human cerebellum
Dang et al. [120]	Mouse	scRNA-seq	Mouse model of SHH	GSE166691	TAMs heterogeneity in SHH MBs and increased TAM subpopulation after radiation along with decreased T cells and neutrophils infiltration
Vo et al. [126]	PDOX	ST-seq	PDOX model of SHH	https://www.ebi.ac.uk/biostudies/arrayexpress/studies/E-MTAB-11720	CDK4/6 inhibition treatment with Palbociclib leads to reduced cellular heterogeneity and higher levels of neuronal differentiation within tumors
Luo et al. [103]	Human	scRNA-seq snATAC-seq	SHH, G3, G4, Human cerebellum	GSE198565; EGAD00001004435; CNP0002781	TCP is a putative cellular origin for Group 3 MBs and tumor-driver networks within TCP-like cells as the potential therapeutic avenues

*MFNs* mossy fiber neuron, *GNPs* granule neuron progenitors, *GlutaCN* glutamatergic cerebellum nuclei, *UBCs* unipolar brush cells, *TCPs* transitional cerebellum progenitors, *RL*^*VZ*^ rhombic lip ventricular zone, *RL*^*SVZ*^ rhombic lip subventricular zone, *TAMs* tumor-associated macrophages, *PDOX* patient-derived orthotopic xenograft, *ST-seq* spatial transcriptomics sequencing, *snATAC-seq* single-nuclei assay for transposase-accessible chromatin with sequencing.

The heterogeneities of cellular components and tumor origin of MBs have been uncovered to a large extent utilizing the single-cell technologies. However, the heterogeneities between primary and recurrent or metastatic MBs at single-cell level are still not fully understood. Relapse is the leading cause of death in patients with MBs, which occur in approximately 30% of patients and are almost always fatal with less than 5% of patients surviving. Morrissy et al. have demonstrated that recurrent MB is highly genetically divergent from matched primary MB, and the genetic divergence with loss of targets at recurrence could account for failure in clinical trials [127]. Hill et al. revealed that combined *MYC* family amplifications and P53 pathway defects commonly emerged at relapse and all patients in this group died of rapidly progressive disease post relapse [128]. Borgenvik et al. indicated that SOX9^+^ quiescent cells accumulated and facilitated MYC-driven recurrence of MBs [129]. Hill et al. revealed that group 3 MB patients relapsed significantly more quickly than did patients with group 4 MBs and distant relapse was prevalent in patients with group 3/4 MBs [130]. For MB metastasis, Wu et al. revealed that metastases from an individual were genetically similar to each other but were divergent from the matched primary MB. Metastases arise from a restricted subclone of the primary MB through clonal selection in both mouse model and patient MBs [131]. Ramaswamy et al. showed that local recurrences were more frequent in SHH MBs and metastatic recurrences were more common in group 3/4 MBs [20]. Fults et al. indicated that group 3 MBs have the highest incidence of metastasis at initial diagnosis and recurrence, whereas WNT MBs exhibited the lowest [132]. Therefore, a deeper understanding of heterogeneities between primary and recurrent or metastatic MBs at single-cell level may help to develop more targeted therapeutic strategies.

Although scRNA-seq is a powerful tool that can be used to dissect intra- and intercellular heterogeneity and tumor origins at the single-cell level, scRNA-seq alone cannot provide spatial information and the tumor microenvironment heterogeneity from in situ location. The ST-seq technology can address the limitation of scRNA-seq by providing whole transcriptome analysis across intact tissue sections without the need to dissociate cells from their in situ localization. The ST-seq has been used in a wide range of tumors to dissect the in situ information, such as glioblastoma. However, the MB patient tissues have yet not been explored using the advantage of ST-seq technology. The spot analysis is the main method used in ST-seq technology. A Visium spot often contains multiple cells, which limits its usage in resolving detailed tissue structure and in characterizing cellular communications. Lack of single-cell resolution in current ST-seq technology may lead to lose of critical information and limit its extensive application [133]. Besides the low spatial resolution, other aspects, such as RNA capture efficiency, data registration across slices, elimination of batch effects and data normalization, remain to be improved. To combine the powers of scRNA-seq and ST-seq to best study tumor biology, the scRNA-seq datasets are usually integrated as a reference for deconvolution of ST-seq datasets, which can partially resolve the issue of low resolution of ST-seq. The ST-seq at single-cell resolution will no doubt lead to new insights and the development of new therapeutics in the next decade. In addition, the current single-cell and spatial technologies are mainly focused on transcriptomic profile. Whereas the epigenome, methylome, proteome and metabolome at single-cell level combined with spatial technology may provide inspiring advance for understanding the heterogeneity of MB in the future.

## Data Availability

All data are included in the published review manuscript.
